# Are Molecular Alterations Linked to Genetic Instability Worth to Be Included as Biomarkers for Directing or Excluding Melanoma Patients to Immunotherapy?

**DOI:** 10.3389/fonc.2021.666624

**Published:** 2021-05-05

**Authors:** Giuseppe Palmieri, Carla Maria Rozzo, Maria Colombino, Milena Casula, Maria Cristina Sini, Antonella Manca, Marina Pisano, Valentina Doneddu, Panagiotis Paliogiannis, Antonio Cossu

**Affiliations:** ^1^ Institute of Genetic and Biomedical Research (IRGB), National Research Council (CNR), Sassari, Italy; ^2^ Institute of Biomolecular Chemistry (ICB), National Research Council (CNR), Sassari, Italy; ^3^ Department of Medical, Surgical, and Experimental Sciences, University of Sassari, Sassari, Italy

**Keywords:** melanoma, microsatellite instability, aneuploidy, tumor mutation burden, immunotherapy response

## Abstract

The improvement of the immunotherapeutic potential in most human cancers, including melanoma, requires the identification of increasingly detailed molecular features underlying the tumor immune responsiveness and acting as disease-associated biomarkers. In recent past years, the complexity of the immune landscape in cancer tissues is being steadily unveiled with a progressive better understanding of the plethora of actors playing in such a scenario, resulting in histopathology diversification, distinct molecular subtypes, and biological heterogeneity. Actually, it is widely recognized that the intracellular patterns of alterations in driver genes and loci may also concur to interfere with the homeostasis of the tumor microenvironment components, deeply affecting the immune response against the tumor. Among others, the different events linked to genetic instability—aneuploidy/somatic copy number alteration (SCNA) or microsatellite instability (MSI)—may exhibit opposite behaviors in terms of immune exclusion or responsiveness. In this review, we focused on both prevalence and impact of such different types of genetic instability in melanoma in order to evaluate whether their use as biomarkers in an integrated analysis of the molecular profile of such a malignancy may allow defining any potential predictive value for response/resistance to immunotherapy.

## Introduction

The increasing efficacy of immunotherapy with immune checkpoint inhibitors (ICIs) has deeply changed life expectancy for different types of fatal cancer: melanoma, lung cancer, renal carcinoma, advanced squamous cell carcinoma of the head and neck or skin districts, some colorectal cancers, and refractory lymphomas (1–5). At the same time, it is widely recognized that the therapeutic indication of ICI cannot be extended to all subtypes of tumor histology since it has been observed that majority of patients are not responsive (6). Therefore, the identification of biomarkers able to accurately predict either response or resistance to the treatment represents a crucial need in cancer immunotherapy.

Although the introduction into clinical practice of validated immuno-oncological biomarkers is currently limited by the heterogeneity of the types of specimens analyzed, because of the diversity of the used methodologies and the absence of a real sharing of the produced data, it is necessary to continue to support the efforts in conducting biomarker-driven trials (7). In recent years, multidisciplinary approaches have significantly increased the quest for an even more accurate molecular classification through the assessment of the mutational status in multiple oncogenes and tumor suppressor genes; in the immuno-oncological field, such efforts have already produced some approved tests (PD-L1 expression and microsatellite instability rates) and other advanced tests yet to be fully proven for efficacy (tumor mutation load, neoantigen pattern, intratumor T-cell infiltration rate) (5, 8–10).

Toward a holistic approach aimed at implementing precision oncology for treatment of “difficult” human cancers, should evaluation of genetic instability be included into the patients’ molecular classification, probably even for the cancer types—like cutaneous melanoma—with a recognized low prevalence of such an alteration? In supporting a positive answer to this question, it has been recently demonstrated that a detailed tumor molecular profiling with identification of all low-frequency actionable alterations in pancreatic cancer—a definitely difficult-to-treat tumor—may produce a significant benefit from receiving a matched therapy (11). Before moving in this sense, we retain to firstly go through the features bringing to the classification of an unstable genome.

## Genetic Instability

**Figure 1:**
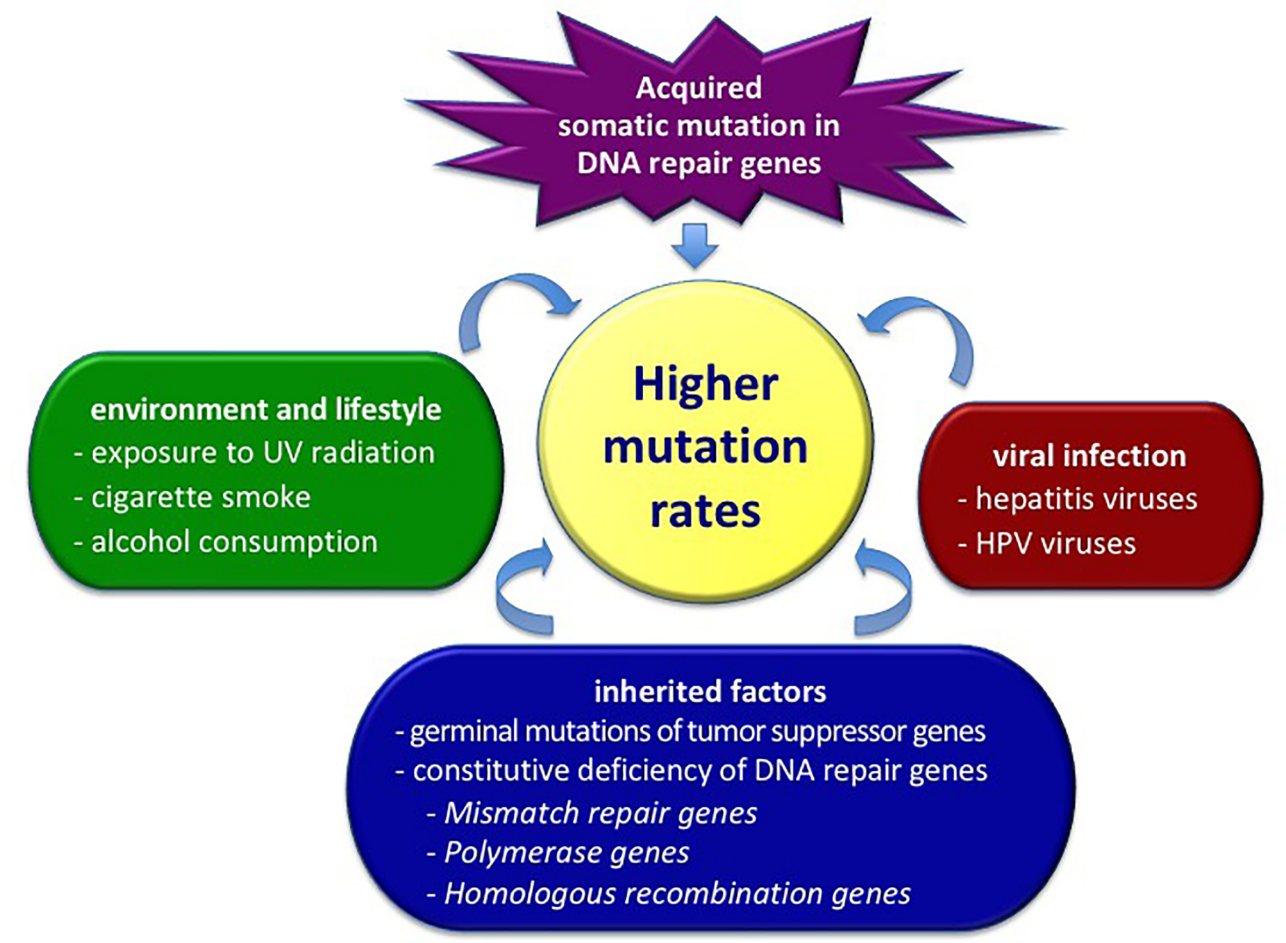
Factors determining the total level of mutations in cancer cells. HPV, human papilloma virus; UV, ultraviolet radiation.

The accumulation and fixation of mutations into the genome, both in the transcribed or regulatory sequences and in those apparently inactive, is one of the most important ways through which evolution is carried out (12). Excluding mutations having deleterious effects with functional consequences, the great majority of sequence variants often display an undefined role (neither harmful nor beneficial) in disease pathogenesis (13). These apparently neutral genetic variants can spread and become fixed in a population, making a large contribution to the evolutionary change in genomes. Focusing on single individuals, the establishment of germinal mutations or the accumulation of somatic mutations can lead to serious cell dysfunctions. **Figure 1** represents the main mechanisms inducing the increase of the mutations’ content in cancer cells.

An accurate and articulated system of control and repair of genomic DNA integrity has evolved into the cells (14, 15). The DNA damage can be caused by genetic instability that may exist at two distinct mechanistic levels. In most cases, genomic instability is observed at the chromosomal level as whole chromosome or segmental/focal aneuploidy; in a more limited fraction of tumors, instability is observed at the nucleotide level and is revealed by the presence of alterations in particular highly repeated DNA sequences with a uniform nucleotide composition, the satellite DNA loci (16, 17). Such satellite DNA regions are classified as minisatellite or microsatellite DNA, depending on the length of the repeated sequences (18–21). Minisatellites consist of repetitive motifs that range in length from 10 to over 100 base pairs. They are located mainly at the centromeres and at the sub-telomeric and telomeric chromosome regions (telomeres itself are constituted by tandem repeats). Minisatellites may play a role in modifying levels of transcription, alternative splicing, or imprinting changes; therefore, they can participate in cell functioning as regulators of gene expression (18, 19, 22). Microsatellites consist of tandem repeats of 1 to 6 base pairs, often organized in long strings, which are subject to mutational events such as insertions and deletions (18, 19, 21).

Aneuploidy—which is due to a genomic imbalance in terms of gain or loss of chromatid or chromosome regions—can be actually classified as a somatic copy number alteration (SCNA), being demonstrated to play a critical role during the process of tumorigenesis and prognosis (23). Occurrence of aneuploidy/SCNA seems to contribute to immune evasion through the reduction of a cytotoxic immune infiltrate into the tumor microenvironment (TME); on this regard, TME can be immunosuppressive *per se*, facilitating tumor progression through mobilization of cytokines, chemokines, and inhibitory factors (24). Moreover, the TME can also recruit immunosuppressive immune cells including regulatory T cells (TREGs), myeloid-derived suppressor cells (MDSCs), and tumor-associated macrophages (TAMs) to evade immune clearance (25). The aneuploid status may potentiate the immunosuppressive TME activity by also negatively interfering with the presentation of the antigens of the major histocompatibility complex (MHC), which represents a fundamental moment into the recognition of the tumor by the immune system (26). The content of peptide neoantigens seems to vary based on the levels of tumor SCNAs, with a relative concentration that is significantly lower in aneuploid tumors than diploid ones acting in an opposite way from the increased overall mutation load and correspondent tumor neoantigen expression levels, which are both positively correlated with the induction of cytotoxic immune infiltrates (27).

Microsatellite instability (MSI) seems to be usually due to deficient DNA damage repair; it has been associated with promotion of a higher load of tumor mutations (28, 29). The MSI occurrence (MSI+) is subsequent to impairment of at least one main gene regulating the different DNA repair mechanisms: homologous recombination (involving *BLM*, *BRCA1/2*, *BRIP1*, *PALB2*, *RAD50/51*, Fanconi Anemia genes), mismatch repair (*MLH1*, *MSH2*, *MSH6*, *PMS2*), cell cycle checkpoints (*ATM*, *CHEK1/2*), base excision repair (*POLE*) (30, 31). A high tumor mutation burden (TMB-high) is generally defined as the >10–20 mutations per megabase of genomic area (threshold is deeply varying according to the cancer type) and can somehow act as a surrogate marker of the neoantigen load (32–34). Tumor specific peptide epitopes, which are usually absent in the normal human genome, can be recognized and targeted by the immune system (33–35). Both MSI+ and TMB-high have been both associated with favorable outcome to ICI therapy in some cancer types (33, 34, 36), but their role in predicting overall survival is still controversial. Vast majority of MSI+ samples present with TMB-high (83%), but the converse is not true, since only 16% of samples with TMB-high are classified as MSI+ (37).

**Figure 2:**
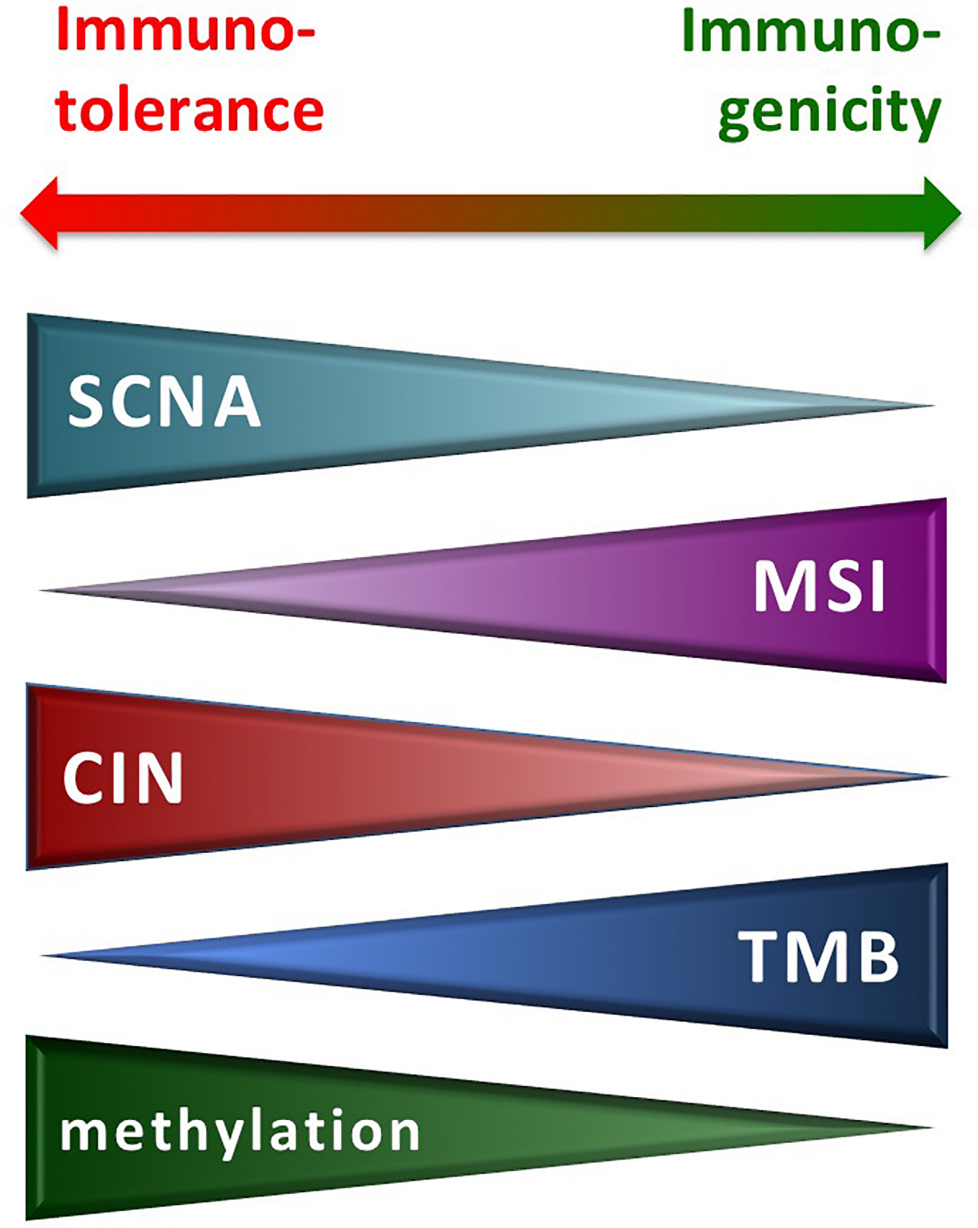
Molecular alterations from genetic instability and immune reactivity. CIN, chromosomal instability; MSI, microsatellite instability; SCNA, somatic copy number alteration; TMB, tumor mutation burden.

Overall, next-generation sequencing (NGS) analysis through a whole genome or exome screening is being used for detecting the high-level SCNAs, the MSI+ status, and the TMB-high in tumor tissues. The MSI+ and TMB-high conditions have been associated with the long-term response to ICI treatment in different human malignancies—including melanoma, lung and renal/bladder cancer, head and neck squamous cell carcinoma (38–45). Conversely, occurrence of aneuploidy/SCNA negatively correlates with the presence of a favorable immune signature, conferring resistance to ICI treatment (26). **Figure 2** summarizes the effects exerted by the different conditions on the activity of the immune system.

Although additional factors are involved in augmenting the adaptive immunity under ICI therapy—such as the histocompatibility leukocyte antigen (HLA) evolution pattern and tumor-infiltrating lymphocyte (TIL) reactivity (27), the simultaneous assessment of the SCNA burden and the rates of TMB and MSI in tumor tissue sections might be strongly useful for classifying patients who are more or less likely to respond to immunotherapies (46). Despite such recognized predictive values, the NGS-based test was not yet routinely included in clinics due to the required high level of technical expertise, the lack of standardization, the high cost, and the pretty-long time required to perform an extensive genomic screening (47, 48). Recently, the combination of reducing the costs of NGS technologies and developing large but manageable multi-gene panels has contributed to facilitate continuous implementations for the use of NGS-based assays in daily clinical practice (49). In other words, the aim of simplifying the sequencing of multiple genes per tumor sample, in order to detect targetable genomic alterations, is becoming a reality and NGS is presenting a really good analytical validity, with an increasingly favorable cost–benefit ratio. To achieve the most currently accurate molecular classification for guiding treatment decisions among cancer patients, recommendations on how multi-gene NGS assays should be used to profile human tumors for improving patients’ management are being provided by scientific societies (50).

### Aneuploidy: Mechanism and Effects

Aneuploidy can be mostly considered as the result of the impairment of the cell cycle checkpoints, which consist of mechanisms that verify DNA replication accuracy and control the cell cycle progression, detecting errors in DNA repair, DNA synthesis, and chromosome segregation (51). Occurrence of structural alterations significantly affecting the genome integrity constitutes a signal sent to the replication/segregation machinery in order to repair the damage (52).

Several cyclin-dependent kinases (CDKs) physiologically drive cell division and regulate the different phases of the cell cycle through phosphorylation of a complex network of substrates and activation of cascades of transduction signals (53). In case of genomic DNA damage, the cell cycle checkpoints arrest the G1/G2 and G2/M transitions by repressing the CDK activity. Hyperactive CDKs, caused by mutations in genes controlling the DNA damage response pathway, lead to the progression into the cell cycle and cell survival (52). On this regard, inactivating mutations in TP53 gene have a permissive role, strongly contributing to the propagation of genetic errors in descendant daughter cells (54). As consequence, deregulation of the TP53-driven pathway—also including impairment of the activity of its downstream effectors (*i.e.*, RB1)—contributes to aneuploidy (55). A number of cancers with mutated TP53 are chromosomal stable and show MSI+, whereas TP53 loss-of-function is predominant in non-hypermutated tumors (54–56). Indeed, the TP53 inactivation is mostly dependent on whether or not mutations in this gene affect the function of p53 on repressing the activity of the Cyclin D1–CDK2 system controlling centrosome duplication and preventing aneuploidy (53).

Activating mutations in oncogenes (such as *CCND1*, *EGFR*, *PIK3CA*, *KRAS*, *BRAF*) and inactivating changes in tumor suppressor genes—like *RB1*, *APC*, and WNT signaling pathway components (*CHK1* and *CHK2*-*BRCA1*)—can dramatically enhance cell proliferation and increase the replication stress levels, causing double-strand breaks in the DNA, with consequent genomic instability that affects tumor progression (57). This seems due to the fact that the unbalanced activity of the driver genes involved in promotion of cell proliferation and survival leads to a sort of oncogene-induced mitotic stress status (58). The enormous variation of segregation errors among different malignancies is indeed a strong indicator that mitotic events act as important players in aneuploidy occurrence (59). Deregulation of the centrosome duplication may indeed promote the formation of multiple centrosomes, which in turn leads to multipolar spindles and aneuploidy (58, 59). Molecular alterations favoring instability of centromeres can thus lead to chromosome segregation defects.

Actually, assessment of aneuploidy is mostly based on measuring SCNA rates in malignancies through bioinformatics approach, the allele-specific copy number analysis of tumors (ASCAT), using data generated by whole-genome/exome sequencing strategies (60). The rates of intratumor karyotype heterogeneity can accurately be determined by simultaneous estimation of the allele-specific total copy number after adjusting for both tumor ploidy—including gains, losses, copy number-neutral events, and loss of heterozygosity (61).

Individual chromosome arm-level alterations were found to be related to expression changes in immune and cell-cycle markers, independent of aneuploidy level; however, increased arm- and chromosome-level SCNA burdens were associated with proliferation signatures and immune evasion profiles (62). Moreover, tumor aneuploidy is likely to increase intratumor heterogeneity, which may inhibit tumor immunity (63). Many solid cancers presenting with a high somatic copy alteration burden exhibit features of immune exclusion, whereas tumors displaying low rates of aneuploidy present an immune active profile (26, 27, 64). High-level SCNAs are classified through bioinformatic approaches as events where focal copy number gain (or loss) are higher (or lower) than the maximum (or minimum) median arm-level copy number gain (or loss), hence avoiding artifacts or false positives after comparison with low-level SCNAs linked to the ploidy of tumor samples and thus obtaining more reliable thresholds (65, 66). High-level SCNA profile in activating beta-catenin signaling pathway elements including *CTNNB1*, *APC*, and *AXIN1-2* genes has been reported in metastatic melanoma but not in primary melanoma (67). A significantly higher concordance between mutated SCNA profiles in beta-catenin signaling pathway activated samples with a low level of T-cell tumor inflammation has been demonstrated, thus suggesting that SCNA signature may act as a progression marker in advanced melanoma (67). For its prediction of the T-cell-inflamed gene expression signature, the SCNA score is worth to be included in molecular tests aimed at somehow anticipating probabilities of resistance to immunotherapies. Further supporting this, the SCNA level has been found lower in lung cancer patients with a responsive disease than those with stable or progressive disease under ICI treatment (68).

Finally, SCNAs can be intrinsically linked to complex structural variants (CSVs) in affecting the efficacy of ICI treatment in melanoma. In particular, CSVs—which are represented by deletions, duplications, translocations, or inversions and arise through the breakage and fusion of one or two genomic locations—are particularly reported in acral melanoma (69). In bioinformatic analysis of NGS-generated data, SCNAs and CSVs are detected as changes in sequencing read depth and in junction-spanning read pairs across the candidate genomic loci (70).

### Microsatellite Instability

MSI is characterized by small insertions or deletions within short tandem repeats in tumor DNA when compared with the corresponding normal DNA. In other words, regions that contain sequences of repeated nucleotides are intrinsically unstable and the insertion of inappropriate nucleotide(s) or the slippage events during DNA replication give rise to the insertion or deletion of single bases or small tandem DNA sequences (56). These alterations, which are normally recognized and repaired, in the absence of an efficient MMR function, are maintained giving origin to alleles of different sizes during the successive replication cycles. The accumulation of unpaired alleles is at the basis of such a genome-wide genetic instability, which is recognized as MSI+ phenotype and observed at higher prevalence in gastrointestinal and endometrial cancers (37, 44, 56, 71). **Table 1** report frequencies of MSI+ in different tumor types, as inferred taking into the consideration the main published studies (72–76).

**Table 1:** MSI+ frequency in different tumor types.

Cancer	Number	MSI+	%
Endometrial carcinoma	1426	401	28.1
Gastric adenocarcinoma	573	117	20.4
Colorectal adenocarcinoma	1,456	196	13.5
Thyroid carcinoma	584	18	3.1
Hepatocellular carcinoma	375	11	2.9
Kidney renal clear cell carcinoma	278	6	2.2
Cutaneous melanoma	359	7	1.9
Ovarian carcinoma	63	1	1.6
Prostate adenocarcinoma	463	3	0.6
Lung nonsquamous cell adenocarcinoma	480	3	0.6
Head and neck squamous cell carcinoma	506	3	0.6
Lung squamous cell carcinoma	443	2	0.5
Urothelial carcinoma	253	1	0.4
Glioblastoma	262	1	0.4
Glioma	513	1	0.2
Kidney papillary cell carcinoma	207	0	0.0
Breast carcinoma	266	0	0.0
**TOTAL**	**8,507**	**771**	**9.1**

Total numbers and percentages were obtained summing data from literature (see text for references).

In colorectal carcinoma (CRC), the MSI+ phenotype has been long evaluated for its impacts on disease pathogenesis and behavior as well as for correlations with prognostic effects. While some distinct clinical and pathological features (proximal location, poor differentiation, mucinous histology) have been consistently associated with the occurrence of MSI, more controversial data have been produced on the prognostic role of this alteration (77). In early stage CRC, the MSI+ phenotype has been described in patients with a better prognosis; conversely, detection of unstable microsatellites seems to confer a negative prognosis in patients with metastatic disease (77–79).

MSI reflects a defect in genes involved in DNA replication fidelity and mostly, is due to inactivation of the mismatch repair (MMR) genes (29, 31). The MMR genes may be impaired by inactivating or down-regulating genetic mutations as well as by gene-silencing epigenetic changes (80). The result of such alterations is the expression of normal levels of functionally deficient MMR proteins or lack of the MMR protein expression, both conditions progressively inducing genetic instability and somehow providing a selective advantage during neoplastic transformation and progression (80). The important components of the DNA mismatch repair system are represented by seven specific ATP-binding proteins that work coordinately in sequential steps to initiate repair of DNA mismatches in genomic DNA: MLH1, MSH2, MLH3, MSH3, MSH6, PMS2, and PMS1 (81). Inactivation of MLH1 and MSH2 was detected in more than 85% of the MSI+ tumors (80, 81). Nearly all MMR genes contain a mononucleotide repeat and thus represent the first target of inactivating mutations when the MSI+ phenotype coexists (71).

The real breakthrough in defining a more impacting role of the MSI in the clinic practice for the management of neoplastic patients has been registered in 2017, when the U.S. Food and Drug Administration (FDA) granted approval of an immune checkpoint inhibitor (the anti-PD-1 pembrolizumab) for treatment of patients with cancers carrying MSI or deficient-MMR (82). The approval by FDA of the anti-PD-1 treatment for all advanced MSI+ solid tumors still represents the first regulatory authorization based exclusively on the use of a specific biomarker, regardless of the anatomic location in the body where the tumor originated (“tumor agnostic”) (83). The MSI and the mutation load underlie the response to PD-1 blockade immunotherapy in deficient-MMR human tumors; the extent of response seems to be particularly associated with the accumulation of insertion-deletion (indel) mutational load (84). In a recent meta-analysis of patients with MSI+ cancer, the ICI treatment was significantly confirmed to be associated with high activity independent of tumor type and drug used and MSI status assessment may have a predictive value for the selection of patients to be addressed to immunotherapy (85).

Epigenomic studies have shown that tumors with MSI exhibit hypermethylation of key genes implicated in tumor development (75, 86). The hypermethylated promoters were identified in some genes that regulate some main molecular signaling cascades (75, 76, 87): WNT (in the absence of WNT-signals, *β*-catenin—a key downstream effector of this pathway—is targeted for degradation through phosphorylation; the WNT signals thus stabilize the intracellular levels of *β*-catenin and subsequently increase transcription of downstream target genes in many human cancers), hedgehog (essential for embryonic and postnatal development, this pathway remains in the quiescent state in adult tissues but gets activated upon inflammation and injuries), and PTEN (its inactivation through mixed genetic/epigenetic mechanisms results in persistent activation of PI3K effectors, with an important impact on cell proliferation, apoptosis resistance, angiogenesis, metabolism regulation, genomic instability, cellular senescence, and cell migration). The hypermethylated status is also tightly correlated with the occurrence of somatic mutations in *BRAF* oncogene, overall causing a strong inhibition of the senescence mechanisms and a consequent promotion of an uncontrolled cell proliferation and survival (88, 89). Hypermethylation has also been related to the facilitation of tumor escape by repressing transcriptional expression of interferon (IFN) regulatory factors (90). Indeed, demethylating agents and histone deacetylases are being combined with ICI treatments in numerous clinical trials and types of malignancies (91, 92).

Several additional factors, other than those mainly underlying MSI, have been shown to be involved in determining a hyper-mutated status, such as inactivating mutations in the DNA polymerases as well as exposure to external (cigarette smoke, UV radiation, chemicals) and endogenous (reactive oxygen species) mutagens (93, 94). The hypermutated condition may be related to driver mutations in the DNA *polymerase ϵ* (*POLE*) and *δ1* (*POLD1*) genes among different tumor types, including colorectal, endometrial, and other cancers such as melanoma and lung cancer (95, 96). Deleterious mutations in *POLE*/*POLD1* genes compromise proofreading of genomic DNA during cell replication and the timing of their onset may vary, with constitutional defective MMR followed by acquired secondary *POLE*/*POLD1* defects or *vice versa* (97). It has been shown that the presence of mutations in *POLE* may promote a high level of non-synonymous single-nucleotide variations (ns-SNVs), not tightly associated with the presence of a MSI+ phenotype (the highest mutation rates were observed in MSS tumors) (71). The *POLD1* gene has been found silenced in several cancer types—mostly, in conjunction with a defective *POLE* gene—with increased genome instability and DNA damage effects (98–100). POLD1 is involved in different forms of DNA repair induced by exposure to mutagens, including nucleotide excision repair, double strand break repair, base excision repair, and mismatch repair (101). The coexistence of MSI+ and mutated *POLE* may be associated with higher densities of CD8+ TILs, PD-1-expressing CD8+ TILs, and tumor-infiltrating immune cells with a Th1 phenotype in the TME, strongly predicting response to checkpoint inhibitors (102).

As mentioned above, tumors with the hypermutated status present similar sensitivity to ICI. Indeed, a strong correlation was found between increased load of non-synonymous mutations and clinical benefits to PD-1 inhibition in non-small cell lung cancer (39) or to cytotoxic T-lymphocyte antigen T 4 (CTLA-4) blockade in melanoma (103). Considering such reported outcomes, one can speculate that increased production of neoepitopes predicting response to ICI might be even generated in cohorts of patients with low (<10% of case) or very low (<1%) prevalence of MSI (**Table 1**).

The hypermutated status can be actually defined with more extensively detailed approaches such as NGS or mass spectrometry assays (104). Among strategies not requiring to match normal DNA material, the single-molecule molecular inversion probe (smMIP) assay is able to detect the existence of an impaired intracellular capability of correcting smMIP-induced errors (105). All these screening strategies are useful in a research context, but technically difficult to translate into clinical practice for routine diagnostic application, since either requiring an extensive bioinformatics analysis of the obtained results either remaining still expensive methods (48—50). Conversely, a simple method to directly detect MSI on formalin-fixed paraffin embedded tumor tissue sections is represented by the Idylla™ test, a fully automated PCR-based assay including a high-resolution melting curve analysis. The Idylla™ MSI test is able to detect mutations in seven tumor-specific MSI loci (ACVR2A, BTBD7, DIDO1, MRE11, RYR3, SEC31A, and SULF2), not requiring the analysis of paired normal tissue samples. For more extensive and detailed information about the methodologies aimed at investigating the MSI status, one can refer to the recent report from our group (106, 107).

The contextual assessment of the MSI+ phenotype and the hypermutated status may be strongly indicative for the existence of a higher tumor immunogenicity, though none of the alterations described as immediate biological effects of the MSI+ phenotype and the hypermutated status—the mutation load, the neoantigen prediction, and the intratumor immune cell infiltration rate—may be considered as a reliable predictor of response to anti-PD-1 treatment (108). Several additional molecular factors are suggested to be involved in immune response. Occurrence of mutations inactivating *JAK1*—within the JAK-STAT pathway that regulates different cellular processes—has been reported to confer resistance to the anti-PD-1 treatment by reducing both the PD-L1 expression and the ability to promote the IFN-γ driven response (109, 110). The relationship between such *JAK1* mutations and MSI status is however complex. In patients with tumors characterized by a low prevalence of MSI—including cutaneous melanoma, invasive breast cancer, and prostate adenocarcinoma—deleterious *JAK1* mutations are associated with unfavorable prognosis (109, 110). In MSI+ tumors, *JAK1* silencing seems to instead impair the tumor growth, playing a positive prognostic role (109, 110). This further confirms that often the same molecular alterations occurring in different tumor types have a distinct impact on biological behavior according to the different genetic backgrounds.

### Classification of Melanoma Patients for Genetic Instability

**Figure 3:**
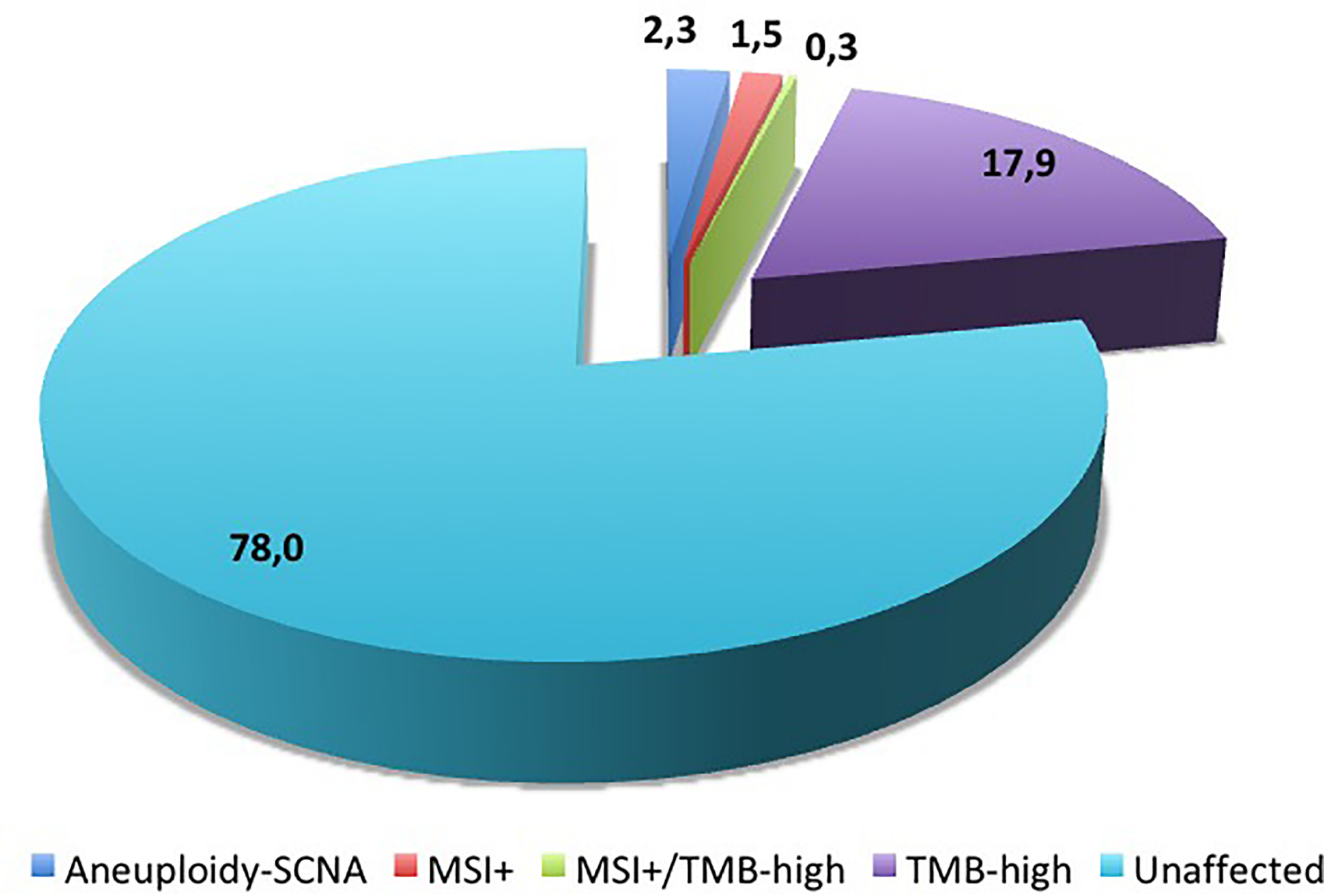
Distribution of molecular alterations linked to genetic instability in melanoma samples. Numbers indicate the percentages of cases reported in literature (see text for references).

According to their mutational status inferred by NGS analysis at somatic level, one could classify melanoma patients using:

-“qualitative” parameters, aimed at discriminating all classes of sequence changes or structural alterations (non-synonymous single-nucleotide variants/ns-SNVs, indels, copy number variations/CNVs, fusions, and splice variants) in tumor suppressor genes and/or oncogenes. These alterations occur at high frequency in melanoma samples. Research efforts should be aimed at defining the clinical role of the distinct mutational patterns of driver ns-SNVs as well as whether the increased load may rather represent the consequence of the sequential accumulation of “passenger” mutations in specific pathways during disease progression;
-“quantitative” parameters, aimed at defining the above described threshold-depending parameters representing the main immuno-oncology content (SCNA, MSI, and TMB). These alterations occur at low frequency in melanoma samples (**Figure 3**).

Most of such key features are actually achieved using large NGS-based panels, which usually include over 400 unique driver genes in correspondent genomic loci for the achievement of a comprehensive and simultaneous genomic profiling (**Table 2**).

**Table 2:** Molecular alterations underlying genetic instability useful in cancer patients’ stratification for immunotherapy.

Type	Detection method	Identified alteration
SCNA	whole genome sequencing (WGS)	gene/locus gain or loss
	whole exome sequencing (WES)	copy number variation
	targeted multiple-gene NGS assays (panels)	complex structural variants
		loss of heterozygosity (LOH)
MSI	Bethesda panel assay (5 microsatellite loci)	genome-wide instability
	*≥ 2 unstable markers (different microsatellite*	
	*lengths between tumor and normal samples)*	
	extended Bethesda panel (8 microsatellite loci and 2 homo-polymer markers: BAT25, BAT26, BAT40, D5S346, D17S250, D2S123, TGFB, D18S58, D17S787, D18S69 or BAT25, BAT26, BAT40, D2s123, D10s197, D13s153, D17s250, D18s58, D5s346, MycI)	genome-wide instability
	*≥30% unstable markers*	mutations in seven MSI loci (ACVR2A, BTBD7, DIDO1, RYR3, MRE11, SEC31A, and SULF2)
	real-time PCR by Idylla™ MSI Test	
	*≥ 1 mutated locus*	
dMMR	protein expression by immunohistochemistry	lack of MMR protein(s)
	targeted multiple-gene NGS assays	mutations inactivating MMR genes (MLH1, MSH2, MLH3, MSH3, MSH6, PMS2, PMS1)
CIN	comparative genomic hybridization (CGH) fluorescence in-situ hybridization (FISH)	whole chromosome or segmental/focal aneuploidy
	gene fusion (mRNA) microarrays	
TMB	whole exome sequencing	mutations per megabase of genomic area
	targeted multiple-gene NGS assays	mutations inactivating DNA polymerases (POLE, POLD1)
Methylation	whole genome methylation	genome-wide DNA methylation with RRBS
	gene promoter methylation	methylation levels of candidate gene promoters

SCNA, somatic copy number alteration; MSI, microsatellite instability; dMMR, deficient mismatch repair; CIN, chromosomal instability; TMB, tumor mutation burden; NGS, next-generation sequencing; RRBS, reduced representation bisulfite sequencing.

### MSI Detection on Liquid Biopsies

In cancer patients, the assessment of PD-L1 status in circulating tumor cells (CTC) and the determination of specific somatic mutations in circulating tumor DNA (ctDNA) represent non-invasive tools acting as predictive markers of the efficacy of the therapeutic response to ICI. The technology for CTC isolation is not widely available, whereas genomic analyzes on ctDNA are methodologically feasible. In NSCLC, undetectable ctDNA levels after two months of ICI were demonstrated to be associated with a marked and lasting response to therapy, while an increase in ctDNA load after initiation of ICI was associated with poorer survival (111, 112). In melanoma, detectable ctDNA at baseline and post-surgical tumour removal may predict a shorter median disease-specific survival among stage III melanoma patients (113, 114) as well as detection of persistent or increasing ctDNA levels during follow-up was shown to predict worse prognosis when compared to patients with undetectable or falling ctDNA levels (115, 116). Currently, plasma-based commercially available assays (“liquid biopsies”) can be used to assess the MSI or the mismatch repair deficiency (dMMR) through genomic analysis by realt-time PCR or DNA sequencing assays in a large variety of cancer types (117–119). From the practical point of view, the real-time PCR is mainly based on the Idylla™ MSI assay (Biocartis, Bruxelles, Belgium; catalog n. A0101/6), which includes a set of seven MSI biomarkers consisting of short homo-polymers located in the above mentioned genes. The NGS tests on ctDNA are performed using complex multigene panels (*i.e*. the Oncomine Comprehensive Assay Plus panel, which provides highly multiplexed target selection of >400 genes implicated in cancer pathogenesis, carried out on the Ion GeneStudio S5 System) (120). These NGS-based tests are now feasible in clinical practice and they have very high concordance, sensitivity and specificity and a detection limit of 0.1% tumor content for MSI-H status. Moreover, such panels allow identification of further genomic alterations (*i.e.* the tumor mutation burden or TMB) with potential implications for predicting response to immunotherapy.

## Conclusive Remarks

Considering the steadily increasing advances in the knowledge of the molecular mechanisms underlying the genetic instability at the chromosomal and nucleotide levels as well as the recognized ascertainment of their clinical impact on cancer management, selection of the subgroups of patients according to the type of instability (SCNA+ *vs.* SCNA−, MSI+ *vs.* MSI−) or mutational composition (TMB-high *vs.* TMB-low; neoantigen-high *vs.* neoantigen-low) present is becoming mandatory. Further advancements will be however achieved by increasing correlations between such molecular features—through a continuous dissemination of the methodologies to be used for their assessment into the clinical practice—and all disease-related and therapy-dependent parameters. These efforts should facilitate the development of innovative diagnostic, predictive, and/or prognostic tools for a better molecular classification of cancer patients, even in a malignancy like melanoma with lower rates of such alterations. Nevertheless, more extensive applications of the NGS technologies could improve the assessment of all driver alterations putatively acting as disease markers to be transferred into the daily clinical practice.

## Author Contributions

All authors contributed to the conception, design, and writing of the manuscript. All authors contributed to the article and approved the submitted version.

## Funding

This work was funded by the Fondazione AIRC “Programma di ricerca 5 per Mille 2018- ID#21073” to EPigenetic Immune-oncology Consortium Airc (EPICA) investigators.

## Conflict of Interest

The authors declare that the research was conducted in the absence of any commercial or financial relationships that could be construed as a potential conflict of interest.
